# Perioperative Pain Management Simulation Course: Improving Anesthesia Trainees’ Confidence in the Management of Perioperative Pain and the Associated Critical Incidents

**DOI:** 10.7759/cureus.49499

**Published:** 2023-11-27

**Authors:** Farooq Afzaal, Pablo R Zamora, Daniel Sciberras, Rhyall Hughes, Lalani K Induruwage, Saurabh Mehrotra

**Affiliations:** 1 Anesthesia, Norfolk and Norwich University Hospital, Norwich, GBR; 2 Anesthesia, James Paget University Hospital, Great Yarmouth, GBR

**Keywords:** simulation in medical education, pain medicine, anesthesia and analgesia, anesthesia training, anesthesia novices, medical education and training, perioperative analgesia, perioperative pain management, perioperative pain, simulation course

## Abstract

Introduction

Pain management is a crucial aspect of patients' perioperative journey and a fundamental duty of every anesthetist. Throughout anesthesia training, there is an emphasis on the management of critical incidents, several of which surround pain management. With changes to the anesthesia curriculum over recent years, variable exposure to training opportunities, and a reduction in clinical hours during training, many trainees report feeling underprepared for their future roles as consultants. However, pain management remains a small fragment of the core anesthesia curriculum with no pain-focused simulation courses currently available across the UK. Simulation has proven to aid learning transfer in complicated and stressful scenarios with a substantial improvement in knowledge retention and prevention of skill loss while eliminating the risk of harm to patients.

Aim

A novel perioperative pain management simulation course was designed and implemented in the East of England to equip junior anesthesia trainees with the knowledge, skills, and confidence to manage perioperative pain and the associated critical incidents.

Methods

A multidisciplinary team (MDT) was involved in the course design. The faculty consisted of anesthesia consultants, trainees, pain nurses, and simulation technicians. The course ran twice over a six-month period both locally and regionally. A blended learning approach was adopted where 17 trainees attended PowerPoint presentations providing an overview of basic pain theories, perioperative pain management, regional anesthesia, and labor analgesia. Trainees then underwent telecasted simulation training using replicated patient notes, imaging, blood gas analysis, and a high-fidelity SimMan®. A debriefing period followed each scenario using Pendleton's model. An anonymized questionnaire was completed by all trainees before and after the course to assess improvement in their knowledge and confidence levels across four domains covering the management of perioperative pain.

Results

All 17 trainees completed the questionnaire; therefore, the entire dataset was analyzed. The pre-course questionnaire showed that using a scale of zero to 10, the vast majority of trainees reported low levels of confidence (<6/10) in the management of chronic pain during the perioperative period (82%), intraoperative pain management (76%), regional anesthesia (88%), and labor analgesia (65%). Following the simulation training, the results showed an overwhelmingly positive improvement in all 17 trainees’ knowledge and confidence across all four tested domains. All 17 trainees (100%) also showed an improvement in their understanding of local pain protocols. The subjective feedback was positive, highlighting the overall usefulness of the course and that the tailored complexity of each simulation scenario was appropriate to each candidate's prior level of experience. Trainees also reported feeling more confident in starting their anesthesia on-calls.

Conclusion

This novel simulation course is the first of its kind in pain management. It has shown great improvements in trainee confidence in managing perioperative pain and the associated critical incidents. Subjective feedback has also been positively reassuring. Its inclusion into the East of England anesthesia training program and national training curriculum would greatly enhance trainee’s knowledge and experience in pain management in the perioperative setting.

## Introduction

This work was previously presented as an oral presentation at "XXXVI National Congress of SEDAR Sections" on 6 October 2023 and at "NHSE East of England Trainee Autumn Symposium" on 11 October 2023. It is also due to be presented as an oral presentation at "The Faculty of Pain Medicine Annual Congress" on 28 November 2023.

Throughout the perioperative journey, anesthetists are responsible for providing high-quality care to patients in stressful and complex settings. The role of an anesthetist is vast and includes preoperative optimization, provision of intraoperative anesthesia and analgesia as well as postoperative care. At times, they are also involved in the management of patients in the high-dependency or intensive care unit. While pain management remains a crucial part of the patient journey and a fundamental duty of the anesthetist, it constitutes a small fragment of the anesthesia training curriculum with no simulation courses available at present across the UK [1].

The anesthesia training curriculum has seen many changes over the last few years [2]. Growing pressures within the NHS and the introduction of the European Working Time Directive have also greatly impacted the training of doctors within the UK. While many benefits have arisen from these hallmark events, with far fewer hours being spent in clinical practice, trainees are now experiencing a noticeable reduction in exposure to training opportunities. Consequently, trainees have reported feeling underprepared for their roles as consultants in the years to come [3,4].

Simulation training has been a common method to aid learning transfer at undergraduate and postgraduate levels with substantial improvements in knowledge and skill retention reported [5,6]. It has proven to be a highly effective method of learning in various disciplines including obstetric and regional anesthesia by providing a range of high-fidelity scenarios [7,8]. Simulation training replicates an immersive life-like environment to facilitate learning while protecting patients from undue harm and mitigating ethical tensions as well as practical dilemmas [9]. It incorporates adult learning theories such as behaviorism, cognitivism, and constructivism to allow trainees to hone technical and non-technical skills in rare, complicated, and stressful scenarios [10,11].

A perioperative pain management simulation course was designed and implemented at a district general hospital, the first of its kind. The aim was to equip trainees within the region with the knowledge, transferrable skills, and confidence to deal with common perioperative pain management scenarios and the associated critical incidents.

## Materials and methods

A multidisciplinary team (MDT) was involved in the course design with the faculty consisting of the local pain department, anesthesia consultants with experience in obstetric and regional anesthesia as well as anesthesia trainees and simulation technicians. The course was designed locally and was run twice over a six-month period both locally and regionally.

A blended learning approach was adopted where a total of 17 trainees all in stage one of anesthetics training initially attended short PowerPoint presentations. The presentations were carefully developed and tailored to the stage one anesthesia curriculum and delivered by faculty members with specialist interests in the specific domain being covered. The content of these PowerPoint presentations included an overview of basic pain theories, intraoperative pain management, pain management using regional anesthesia, and the management of labor analgesia. Each PowerPoint presentation lasted 30-40 minutes. 

**Figure 1:**
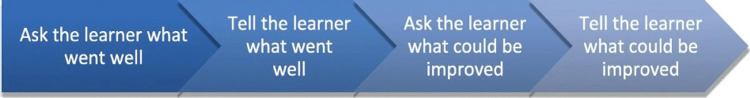
Pendleton's model of feedback

Following this, trainees individually underwent 20-minute telecasted simulation scenarios developed by all faculty members. The scenarios included the use of replicated patient notes, prescription, anesthetic, and intravenous fluid charts as well as radiographical imaging, blood gas analysis results, and a high-fidelity SimMan®. Other paraphernalia included epidural and nerve block catheters as well as c-spine immobilization kits to replicate scenarios involving troubleshooting of nerve blocks and epidurals and pain management of the trauma patient. All the scenarios were telecasted in an adjacent room for the remainder of the trainees to view, allowing for a maximally engaging learning experience and facilitating a platform for constructive feedback from fellow trainees. Faculty members ran the clinical scenarios from inside the simulation room while simulation technicians managed the SimMan® and its responses. All the scenarios were carefully tailored to each trainee’s prior experience level to optimize individual learning. A 10-minute debrief period followed each scenario where constructive feedback was provided using Pendletons’ model by both the faculty and fellow candidates [12] (Figure 1).

All trainees completed an anonymized pre- and post-course questionnaire gathering both qualitative and quantitative feedback. The data gathered included the trainees’ stage of training, their knowledge of local pain protocols, and their understanding of where to seek expert advice relating to pain management. Trainees' confidence levels in various domains surrounding perioperative pain management were assessed both before and after the course using this survey-based methodology. Subjective feedback on the entire course was also obtained through the questionnaires. 

## Results

All 17 trainees completed the survey both before and after the teaching course; therefore, the complete dataset was analyzed. The results of the survey showed an overwhelmingly positive increase in the trainees' confidence levels for all tested domains, and the subjective feedback was encouraging, highlighting the usefulness of the course. The subjective feedback highlighted that the tailored complexity of each simulation scenario was appropriate to each candidate's prior level of experience and that the trainees benefitted from the structured debriefs and felt more confident in being able to start their anesthetic on-calls. As per the quantitative results, all 17 trainees also reported a complete understanding of local pain protocols following the course and reported finding the entire course useful.

**Figure 2:**
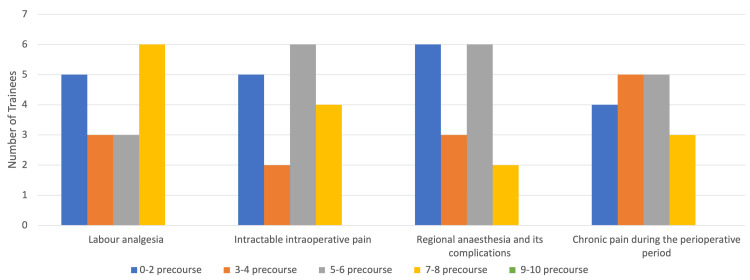
Number of trainees (n) (y-axis) reporting pre-course confidence levels ranging from zero to 10 in all four tested domains (x-axis)

The pre-course survey showed a wide variation in the confidence levels of all 17 trainees in managing labor analgesia, intractable intraoperative pain, regional anesthesia and its complications, and chronic pain during the perioperative period. Confidence levels of less than two out of 10, three to six out of 10, and seven or more out of 10 were considered “not confident,” “somewhat confident,” and “very confident,” respectively. The vast majority of trainees reported confidence levels equal to or below six out of 10 in all domains prior to undergoing simulation training, i.e., 11/17 (65%) for labor analgesia, 13/17 (76%) for intraoperative pain, 15/17 (88%) for regional anesthesia and its complications, and 14/17 (82%) for management of chronic pain during the perioperative period. No trainees felt nine to 10 out of 10 confident in any of the domains prior to the simulation training. These pre-course survey results highlight the poor overall confidence of the majority of trainees in all domains prior to undergoing the simulation experience (Figure 2).

**Figure 3:**
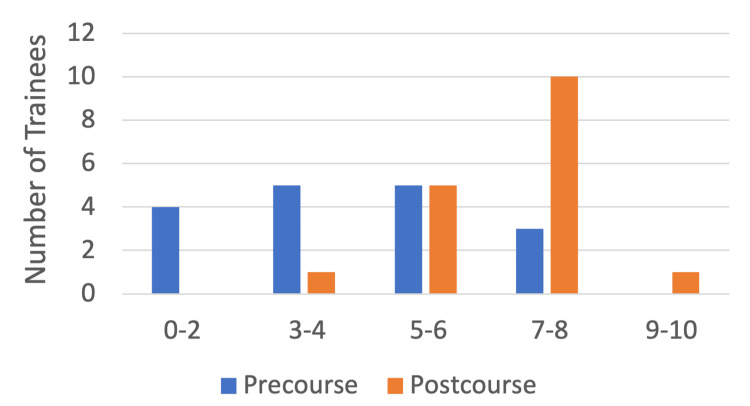
Number of trainees (n) (y-axis) reporting pre- and post-course confidence levels (x-axis) in perioperative chronic pain management

As per the post-course survey results, trainee confidence in the management of chronic pain during the perioperative period greatly improved following the course where confidence levels of greater than seven ("very confident") increased from only three trainees (17%) pre-course to 11 trainees (65%) post-course with one trainee feeling nine to 10 out of 10 confident in this domain following the course. Only six (35%) of the trainees felt "not confident" or "somewhat confident" in this domain following the course while previously 14 trainees (85%) fell in this bracket, showing an overall improvement in this domain through course attendance (Figure 3).

**Figure 4:**
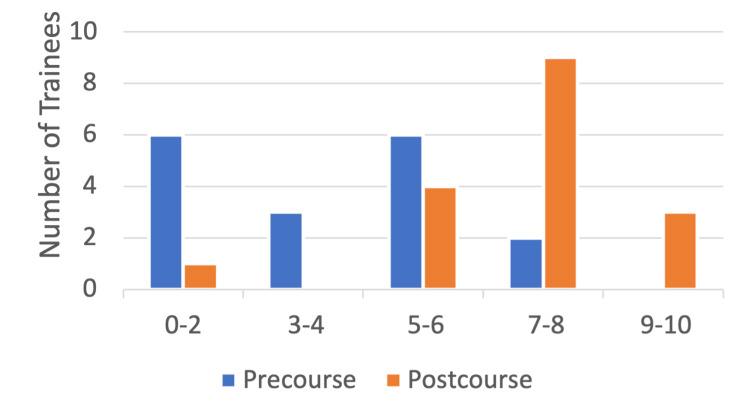
Number of trainees (n) (y-axis) reporting pre- and post-course confidence levels (x-axis) in managing regional anesthesia and its complications

Similarly, trainee confidence of greater than or equal to seven ("very confident") for managing regional anesthesia and its complications increased from only two trainees (12%) pre-course to 12 trainees (71%) post-course while three trainees (17%) felt nine to 10 out of 10 confident post-course. While 15 of 17 trainees (88%) felt "not confident" or "somewhat confident" in this domain prior to the course, only five trainees (29%) fell in this bracket following the course (Figure 4).

**Figure 5:**
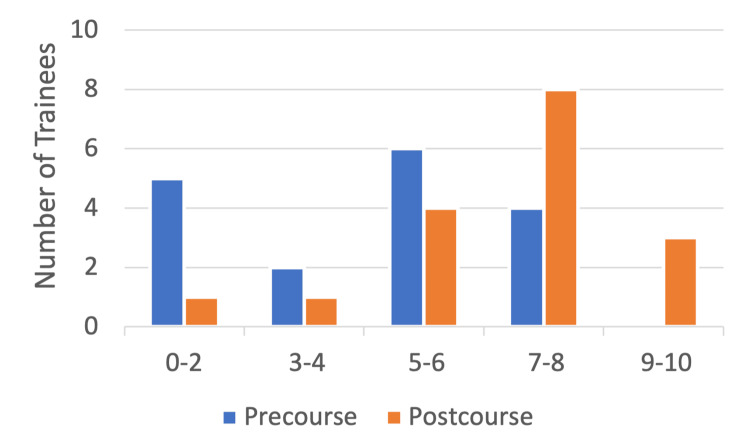
Number of trainees (n) (y-axis) reporting pre- and post-course confidence levels (x-axis) in managing intraoperative pain

Trainees’ confidence level of greater than or equal to seven ("very confident") in managing intraoperative pain improved from four trainees (24%) pre-course to 11 trainees (65%) post-course while three trainees (17%) felt nine to 10 out of 10 confidence in managing intraoperative pain following the course. While 13 of 17 trainees (76%) felt "not confident" or "somewhat confident" in this domain prior to the course, only six trainees (35%) fell in this bracket following the course (Figure 5).

**Figure 6:**
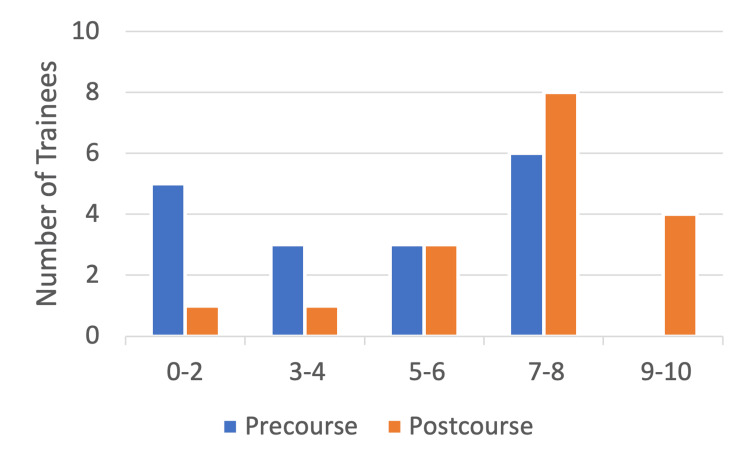
Number of trainees (n) (y-axis) reporting pre- and post-course confidence levels (x-axis) in managing labor analgesia

Finally, trainees’ confidence level of greater than or equal to seven ("very confident") in managing labor analgesia improved from six trainees (35%) pre-course to 12 trainees (71%) post-course while four trainees (24%) felt nine to 10 out of 10 confidence in managing labor analgesia post-course. While 11 of 17 trainees (65%) felt "not confident" or "somewhat confident" in this domain prior to the course, only five trainees (29%) fell in this bracket following the course (Figure 6).

## Discussion

Anesthesia is a craft specialty consisting of numerous pain management procedures that are performed based on a sound understanding of anatomy, physiology, and pharmacology. Extensive experience is required to safely perform these procedures and to confidently manage their various complications. This simulation program was designed to allow trainees to have repetitive exposure to rare, stressful, and unfamiliar pain management scenarios and their associated critical incidents. This was performed in a high-fidelity setting in order to best aid learning transfer while eliminating any risk of harm to patients.

The numerical data and subjective feedback received from the course were overwhelmingly positive and trainees felt notably more confident in all tested domains using the pre- and post-course surveys. All trainees showed a clear improvement in their understanding of local pain protocols including the availability and accessibility of specialist pain nurses and a pain consultant both in and out of hours. This knowledge led to most trainees feeling more comfortable in complex perioperative scenarios as there was an increased awareness of the whereabouts of senior support, particularly during their on-call shifts.

As part of stage one training in the anesthesia curriculum, trainees are expected to be well-versed in labor analgesia and the management of its complications. As junior trainees in the early stages of their careers, many felt inexperienced in managing the various complications that can occur in these circumstances. Having compiled a variety of scenarios including failed blocks, unilateral blocks, patchy blocks, catheter displacements, and high spinal/epidurals, trainees were able to experience these clinical situations in life-like scenarios. Survey results showed a significant improvement in trainee confidence in the management of labor analgesia as well as regional anesthesia and its complications.

The survey results suggest that following the simulation, one to two trainees remained “not confident” in some of the tested domains. This can be explained by the varying experience of the trainees attending the course. While all efforts were made to cater to the candidate’s previous experience, a few trainees were in the very early weeks of anesthesia training with little or no exposure to anesthesia. It is, therefore, completely reasonable for them to remain less confident than others in the management of these complex and stressful scenarios; however, all trainees reported that they found the course useful. 

A limitation of the study is the inability to extrapolate the results beyond the immediate timeframe. In the future, it would be beneficial to repeat candidates’ confidence levels a few weeks after attending the course to assess knowledge and skill retention. A comparison of confidence can also be made with those who have not attended the course within the region. A further limitation of the study is the small sample size due to the logistical difficulties of carrying out simulations of this nature with a large number of candidates over a given timeframe. With each presentation lasting 30-40 minutes, the total duration of the presentations lasted approximately two hours. Following this, each candidate's simulation lasted 20 minutes with a 10-minute debrief period. There were approximately eight candidates in each session, which meant that four hours of simulation and two hours of PowerPoint presentations were required for each training day. This coupled with adequate breaks for the candidates meant that a limited number of candidates could undergo the simulation on each occasion. However, future sessions will obtain a greater sample size of candidates to reduce any possibility of conformity bias and may even allow for statistical analysis of data.

Future developments include the incorporation of an inter-professional approach by inviting fellow disciplines such as operating department practitioners, recovery nurses, and intensive care staff. This skill mix integration will allow the sharing of various perspectives of medical practice with an overall aim of optimizing patient care. The course would also benefit future trainees by being incorporated into the local and regional program for junior anesthetic trainees as well as forming a fundamental component of the core anesthesia curriculum.

## Conclusions

This novel simulation course is the first of its kind in perioperative pain management. The blended learning approach incorporating the effective use of simulation improved the knowledge, skills, and confidence of junior anesthesia trainees in dealing with a variety of perioperative pain management scenarios and their associated critical incidents. The quantitative and qualitative feedback both promote the courses' inclusion into the regional anesthesia training program as well as the Royal College training curriculum.
